# Machine learning and experimental validation of novel biomarkers for hypertrophic cardiomyopathy and cancers

**DOI:** 10.1111/jcmm.70034

**Published:** 2024-08-19

**Authors:** Hualei Dai, Ying Liu, Meng Zhu, Siming Tao, Chengcheng Hu, Peng Luo, Aimin Jiang, Guimin Zhang

**Affiliations:** ^1^ Cardiovascular Center The Affiliated Hospital of Yunnan University, Yunnan University Kunming Yunnan China; ^2^ School of Medicine Yunnan University Kunming Yunnan China; ^3^ Department of Gynecology Yunnan Cancer Hospital and The Third Affiliated Hospital of Kunming Medical University Kunming Yunnan China; ^4^ Department of Geriatrics The Affiliated Huaian Hospital of Xuzhou Medical University, Huaian Second People's Hospital Huaian Jiangsu China; ^5^ Department of Oncology Zhujiang Hospital, Southern Medical University Guangzhou China; ^6^ Department of Urology Changzheng Hospital, Naval Medical University Shanghai China

**Keywords:** biomarkers, hypertrophic cardiomyopathy, immune infiltration, machine learning, pancancer

## Abstract

Hypertrophic cardiomyopathy (HCM) is a hereditary cardiac disorder marked by anomalous thickening of the myocardium, representing a significant contributor to mortality. While the involvement of immune inflammation in the development of cardiac ailments is well‐documented, its specific impact on HCM pathogenesis remains uncertain. Five distinct machine learning algorithms, namely LASSO, SVM, RF, Boruta and XGBoost, were utilized to discover new biomarkers associated with HCM. A unique nomogram was developed using two newly identified biomarkers and subsequently validated. Furthermore, samples of HCM and normal heart tissues were gathered from our institution to confirm the variance in expression levels and prognostic significance of GATM and MGST1. Five novel biomarkers (DARS2, GATM, MGST1, SDSL and ARG2) associated with HCM were identified. Subsequent validation revealed that GATM and MGST1 exhibited significant diagnostic utility for HCM in both the training and test cohorts, with all AUC values exceeding 0.8. Furthermore, a novel risk assessment model for HCM patients based on the expression levels of GATM and MGST1 demonstrated favourable performance in both the training (AUC = 0.88) and test cohorts (AUC = 0.9). Furthermore, our study revealed that GATM and MGST1 exhibited elevated expression levels in HCM tissues, demonstrating strong discriminatory ability between HCM and normal cardiac tissues (AUC of GATM = 0.79; MGST1 = 0.86). Our findings suggest that two specific cell types, monocytes and multipotent progenitors (MPP), may play crucial roles in the pathogenesis of HCM. Notably, GATM and MGST1 were found to be highly expressed in various tumours and showed significant prognostic implications. Functionally, GATM and MGST1 are likely involved in xenobiotic metabolism and epithelial mesenchymal transition in a wide range of cancer types. GATM and MGST1 have been identified as novel biomarkers implicated in the progression of both HCM and cancer. Additionally, monocytes and MPP may also play a role in facilitating the progression of HCM.

## 1 INTRODUCTION

Hypertrophic cardiomyopathy (HCM), the leading cause of sudden cardiac death in adolescents, presents a spectrum of severe complications such as heart failure, stroke and cardiac arrhythmias if not adequately managed.^1^, ^2^ As an autosomal dominant primary cardiomyopathy affecting approximately 0.2% of the population, HCM significantly escalates the risk of developing heart failure—by two to four times compared to the unaffected population—thereby worsening patient prognoses.^3^ Genetic investigations attribute HCM primarily to rare variants within sarcomere‐related genes like MYH7, MYBPC3, MYL2, TNNT2, TPM1 and ACTC1, identified in about 50% of cases.^4^ However, the origins of nearly 40% of HCM cases remain elusive, underscoring a critical gap in our understanding due to the complex relationship between clinical phenotypes and genetic variability.^5^ Historically, research has focused on pinpointing specific mutations, yet this approach often overlooks broader genomic contributions to HCM, particularly in sporadic cases or small familial clusters.^6^ This limitation highlights the imperative need for novel diagnostic and therapeutic targets in HCM management.

Advancements in sequencing technologies and bioinformatic analysis pipelines have led researchers to gain a deeper understanding of omics datasets related to disease progression, resulting in the development of innovative diagnostic and therapeutic models for specific heart diseases.^7^, ^8^ Advancements in sequencing technologies and bioinformatic analysis pipelines have led researchers to gain a deeper understanding of omics datasets related to disease progression, resulting in the development of innovative diagnostic and therapeutic models for specific heart diseases.^6^ Furthermore, the immune system serves a dual function in regulating the normal physiological processes of the steady‐state heart and in modulating adverse inflammatory responses and myocardial remodelling post‐injury. It functions as a surveillance mechanism within the heart, but its activation can also lead to detrimental effects on cardiac tissue.^9^ Prior research has identified potential novel biomarkers and immune components involved in the development of HCM. Wang conducted an analysis using three HCM datasets and identified the potential roles of ferroptosis in HCM, specifically focusing on IGFBP5, FMOD and POSTN.^10^, ^11^ Researchers have identified inflammatory cell infiltration and fibrosis in myocardial tissues of individuals with HCM.^12^ While these findings contribute to a deeper understanding of the condition, limitations such as sample size and lack of external validation may hinder the widespread use of biomarkers. Furthermore, the integration of multiple datasets using machine learning techniques to discover new and reliable biomarkers for HCM is an area requiring urgent exploration.

Despite these advances, challenges such as limited sample sizes and the need for broader validation remain, which can impede the clinical application of new biomarkers. To address these issues, our study integrates multiple omics datasets through advanced machine learning algorithms to robustly identify and validate key biomarkers and to explore immune system variances between HCM and healthy tissues. Additionally, considering the potential link between cardiac diseases and various cancers, our study extends to examine the implications of these newly identified biomarkers across different cancer types, potentially broadening the impact of our findings in the realm of cardio‐oncology.

## 2 MATERIALS AND METHODS

### 2.1 Dataset collection and processing

HCM‐related datasets, specifically GSE130036, GSE141910, GSE160997, GSE36961 and GSE32453, were obtained from the Gene Expression Omnibus (GEO) database using the R package GEOquery. The datasets consisted of varying numbers of HCM and healthy samples.^13^, ^14^ Subsequently, all array datasets were normalized using the normalizeBetweenArrays function within the limma R package. Given the necessity of a sufficient number of samples for machine learning input, we amalgamated GSE130036, GSE141910, GSE160997 and GSE36961 as the training datasets using the R package sva. The expression profiles of solid tumours and clinical outcome data were obtained from TCGA datasets through the R package TCGAbiolinks.^15^ All datasets utilized in this study were sourced from publicly available websites, rendering patient information and ethical approval irrelevant.

### 2.2 Biological difference between HCM and normal tissues

The R package limma was utilized to identify differentially expressed genes (DEGs) between HCM and normal heart tissues, with DEGs defined by abstract fold change >1.2 and *p* value <0.05.^16^ To evaluate the biological relevance of these DEGs, gene ontology (GO) enrichment analysis was conducted using the R package clusterProfiler.^17^ Additionally, potential regulatory interactions between factors and DEGs were investigated through JASPAR and NetworkAnalyst software, with visualization in Cytoscape.^18^, ^19^, ^20^

### 2.3 Identification and verification of novel biomarkers of HCM by machine learning

In our study, we integrated differential expression genes (DEGs) between hypertrophic cardiomyopathy (HCM) and normal tissues to identify novel significant signatures associated with HCM using advanced machine learning algorithms. Specifically, we employed five algorithms: Lasso Logistic Regression (Lasso), Support Vector Machine with Recursive Feature Elimination (SVM), Random Forest (RF), Boruta and eXtreme Gradient Boosting (XGBoost).^21^ Each of these methods plays a critical role in our feature selection process, helping to pinpoint disease diagnostic factors. The Boruta algorithm, a supervised classification feature selection method, was utilized to identify all relevant features essential for the classification task. Lasso is particularly useful for handling categorical variables, enhancing both the predictive accuracy and interpretability of our statistical models. SVM is instrumental in the classification, regression, and feature selection of multi‐class data, effectively managing both homogeneous and heterogeneous features. XGBoost, known for its interpretability, calculates and ranks importance scores for each feature, assessing the contribution of each by the improvement metric in the model's performance. Similarly, RF, a robust ensemble tree‐based method, is highly adaptive to data, elucidating correlations and interactions among features. To assess the effectiveness of these biomarkers in distinguishing HCM from normal tissues, we retained those demonstrating satisfactory performance across all algorithms, evidenced by an area under the curve (AUC) value exceeding 0.65. Subsequently, these selected biomarkers were used to construct a logistic regression model. The dataset from GSE32453 served as the testing cohort, providing a robust framework to evaluate the risk model's accuracy and reliability in a clinical setting.

### 2.4 Immune difference between HCM and normal heart tissues

In order to analyse the immune composition of HCM and normal heart tissues, we employed two deconvolution algorithms, namely Xcell and ssGSEA.^22^ The Xcell algorithm, a commonly utilized analytical tool, utilizes transcriptome data to accurately quantify the presence of immune infiltrates in diverse tissues.^23^ This algorithm has demonstrated efficacy in characterizing immune diseases and tumours by offering insights into their respective immune microenvironments. The normalization expression matrix served as the primary input data for deconvolution analysis, wherein the degree of infiltration between HCM and normal heart tissues was assessed using the Kruskal‐Wallis test. The associations of GATM and MGST1 with the level of immune infiltration were determined through the Spearman correlation coefficient from the R package circlize and depicted using ggplot2. Furthermore, to identify the key immune cells associated with HCM, five machine learning algorithms were employed.

### 2.5 Role of GATM and MGST1 across cancers

In this study, cancer samples from TCGA were stratified into two groups based on the expression levels of GATM and MGST1, specifically the top 25% (referred to as high expression groups) and lowest 25% (referred to as low expression groups) GATM and MGST1 subgroups. Subsequently, DEGs between the high and low expression subtypes were identified and annotated using cancer hallmarks from the MSigDB database. Furthermore, the correlation between GATM and MGST1 and various classic cancer‐related signatures, such as immune checkpoint‐related signatures, DNA mismatch repair (MMR)‐related genes and methyltransferase‐related genes, was investigated.

### 2.6 Validation of different expression levels of GATM and MGST1

HCM and normal heart tissues were obtained from patients who underwent cardiac surgery at our institution. mRNA levels were quantified using real‐time reverse transcription PCR following the manufacturer's protocol. Reverse transcription was carried out using RevertAid RT Transcription Kits (Thermo Fisher Scientific, Waltham, MA, USA). The primers used for GATM were as follows: forward: CACTACATCGGATCTCGGCTT, reverse: CTAAGGGGTCCCATTCGTTGT. The primers for MGST1 were as follows: forward: ATGACAGAGTAGAACGTGTACGC, reverse: TACAGGAGGCCAATTCCAAGA. GAPDH was employed as the internal control. We applied the classic calculation formula (2^−∆∆Ct^ method) to compare the expression levels of GATM and MGST1 between HCM and normal heart tissues.

### 2.7 Statistical analysis

All statistical analyses and final visualizations were conducted using R (version 4.2.0) and SPSS software. The predictive accuracy of each signature was assessed through the calculation of the area under the curve using the R package pROC, while the creation of a nomogram and calibration curves was accomplished with the R package rms. The benefit curve was generated using the R package ggDCA. Validation experiments were independently repeated three times in our study. Survival analysis of GATM and MGST1 across multiple cancers was facilitated by the R packages survminer and ggplot2. A significance level of *p* < 0.05 was applied for statistical inference. More analysis details could be seen in our previous work.^24^, ^25^, ^26^

## 3 RESULTS

### 3.1 HCM dataset processing and identification of differentially expressed genes

**FIGURE 1:**
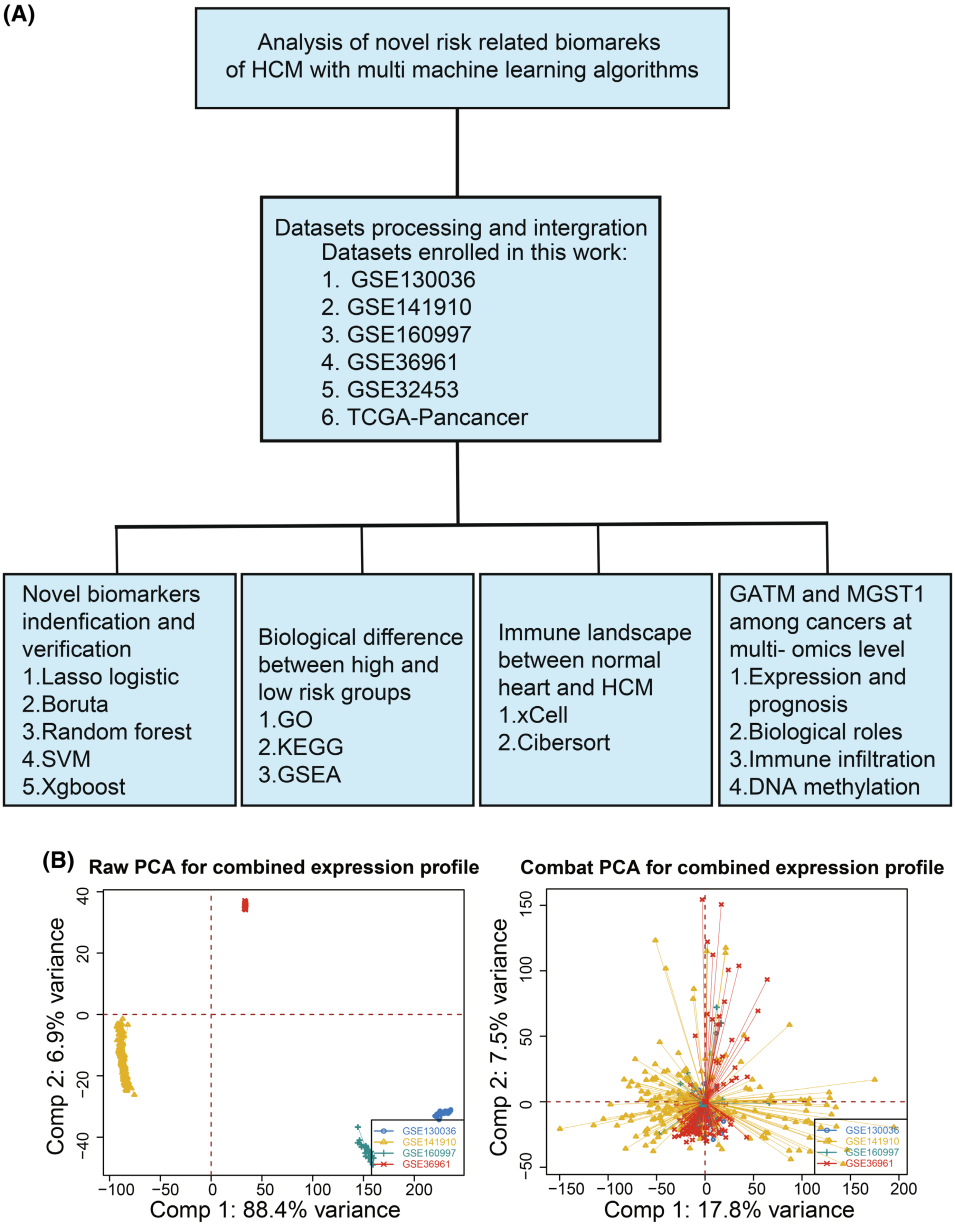
Strategy of identification of novel biomarkers for HCM. (A) Main workflow of this study. (B) PCA plot shows the dataset distribution before (left) and after (right) batch effect removal.

**FIGURE 2:**
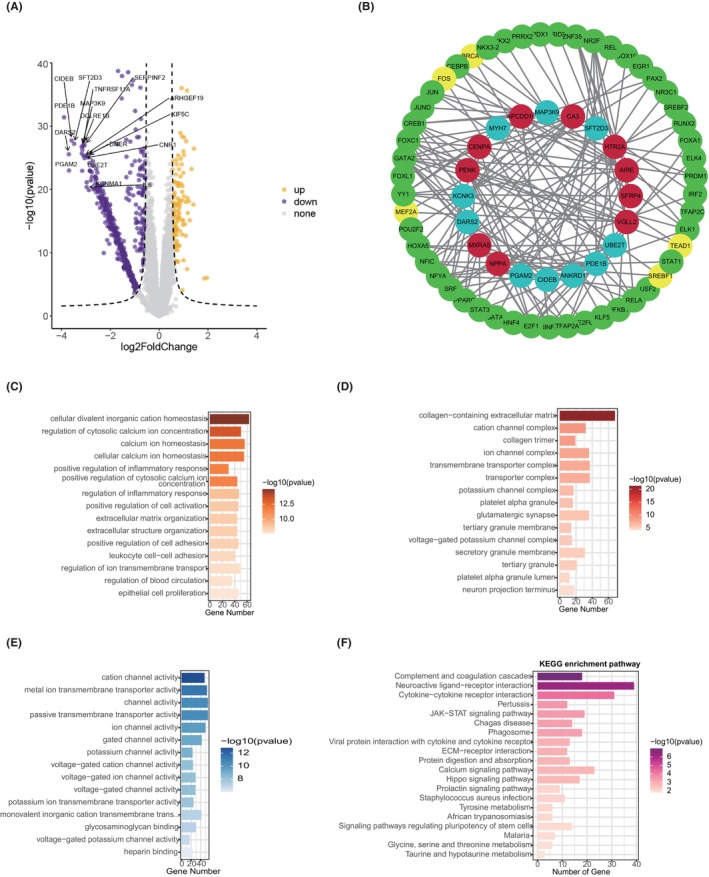
Differentially expressed genes between HCM and normal tissues and corresponding annotation. (A) Volcano plot indicating that orange represents overexpressed genes, while purple represents downregulated genes in HCM tissues. (B) Potential regulatory network between transcription factors and DEGs. Red represents upregulated genes, while blue represents downregulated genes; green stands for TFs, while yellow is the most important TF. (C) Annotation of DEGs in the biological process; (D) cellular component; (E) molecular function and (F) KEGG pathway categories.

The brief workflow of this study is summarized in Figure 1A. The expression profiles of four HCM datasets (GSE130036, GSE141910, GSE160997 and GSE36961) were downloaded and integrated to serve as the training dataset. Figure 1B shows the satisfactory batch effect removal efficacy, and each colour represents an HCM cohort. A total of differentially expressed genes were found between HCM and normal tissues in the training datasets (Figure 2A). We investigated the potential regulatory mechanism and found five leading transcription factors, BRCA1, FOS, MEF2A, SREBF1 and TEAD1 (Figure 2B). We noticed that DEGs between HCM and normal heart tissues might be involved in cellular divalent inorganic cation homeostasis, regulation of cytosolic calcium ion concentration and calcium ion homeostasis in the biological process category (Figure 2C); collagen‐containing extracellular matrix, cation channel complex and collagen trimer in the cellular component category (Figure 2D); and cation channel activity, metal ion transmembrane transporter activity and channel activity in the molecular function category (Figure 2E). Furthermore, we found that the DEGs also participated in complement and coagulation cascades, neuroactive ligand receptor interactions and cytokine receptor interactions (Figure 2F), which suggested that immune processes might play a pivotal role in the progression of HCM.

### 3.2 Novel biomarkers of HCM were identified with multimachine learning algorithms

**FIGURE 3:**
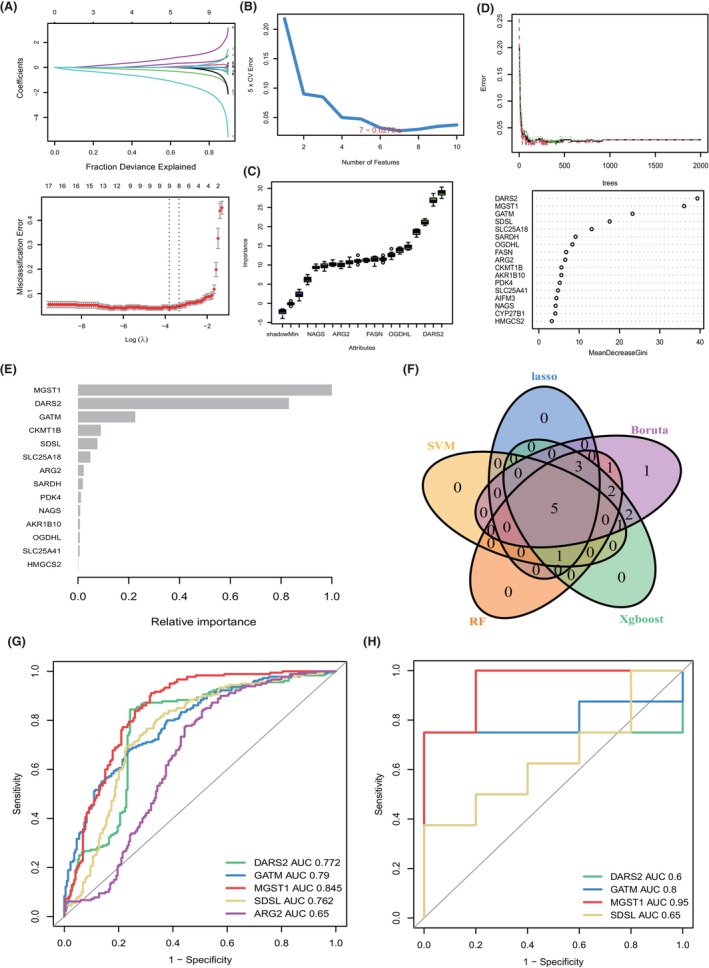
Novel biomarker identification based on machine learning. (A) Biomarkers derived from DEGs were extracted by the LASSO logistic regression algorithm. (B) Future selection based on SVM‐RFE; (C) Boruta; (D) RF and (E) XGBoost in the combined HCM expression matrix. (F) Venn plot showing the interaction of biomarkers from five algorithms. (G) Diagnostic accuracy of biomarkers selected from multimachine learning algorithms in training datasets and (H) testing datasets, which were evaluated by corresponding area under the curves (AUCs).

To investigate novel biomarkers or targets of HCM, DEGs between HCM and normal heart tissues were adopted to perform machine learning algorithms. We used LASSO logistic (Figure 3A), SVM‐RFE (Figure 3B), Boruta (Figure 3C), RF (Figure 3D) and Xgboost (Figure 3E) to identify the most important biomarkers involved in HCM. The intersections of five algorithms are shown in Figure 3F, which included DARS2, GATM, MGST1, SDSL and ARG2. As shown in Figure 3G, five biomarkers reached a satisfactory performance to distinguish HCM from normal heart tissues (AUC of DARS2, GATM, MGST1, SDSL and ARG2 reached into 0.772, 0.79, 0.845, 0.762 and 0.65, respectively). We next chose four biomarkers with AUC >0.7 to validate in the testing cohort and found that GATM (AUC = 0.8) and MGST1 (AUC = 0.95) also reached high accuracy and sensitivity (both AUC >0.8), as shown in Figure 3H.

### 3.3 Risk model based on GATM and MGST1 could serve as a novel diagnostic system

**FIGURE 4:**
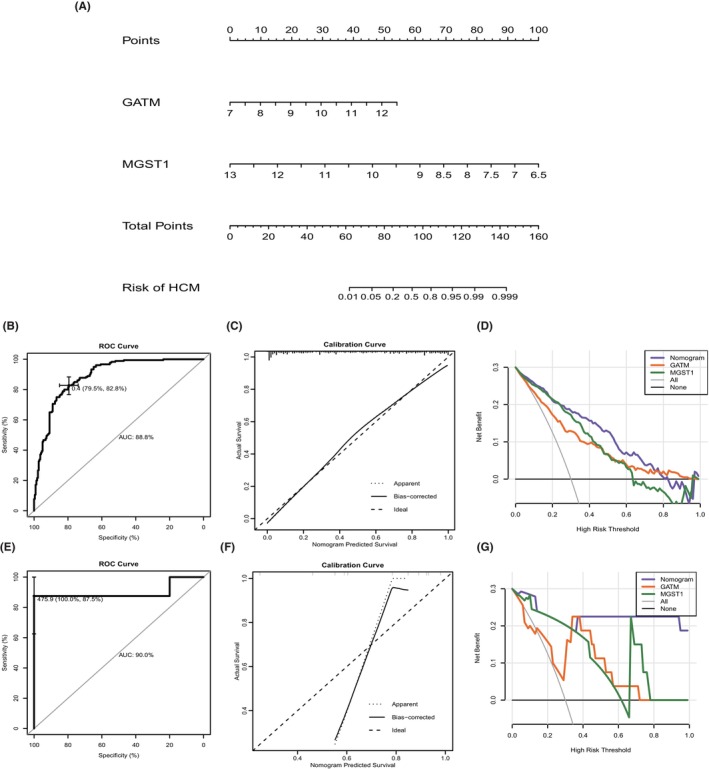
Construction and verification of the novel diagnostic model for HCM. (A) Nomogram based on GATM and MGST1 expression levels. (B) ROC curve, (C) calibration curves and (D) DCA curves to assess the diagnostic and clinical benefit value of the monogram system in training datasets. (E) ROC curve, (F) calibration curves and (G) DCA curves to evaluate the diagnostic and clinical benefit value of the monogram system in testing datasets.

According to the results mentioned above, we noticed that GATM and MGST1 displayed diagnostic value in both the training and testing cohorts. Thus, we chose the two biomarkers to construct a novel quantification system (Figure 4A). After calculating each sample's risk score, we found that the ROC value reached into 0.88, and the calibration curve was also close to the ideal line (Figure 4B,C). The benefit curve also suggested that the combination of GATM and MGST1 could serve better diagnostic value than each gene (Figure 4D). Largely consistent with the findings in the training dataset, the risk scores of GATM and MGST1 also had high accuracy in distinguishing HCM from normal tissues in the testing cohort, with an AUC of 0.9 and a satisfactory calibration curve (Figure 4E,F). According to the benefit curve, we also found that the combination of GATM and MGST1 reached a significant benefit for patients (Figure 4G).

### 3.4 Validation of diagnostic value and investigation of biological roles of GATM and MGST1

**FIGURE 5:**
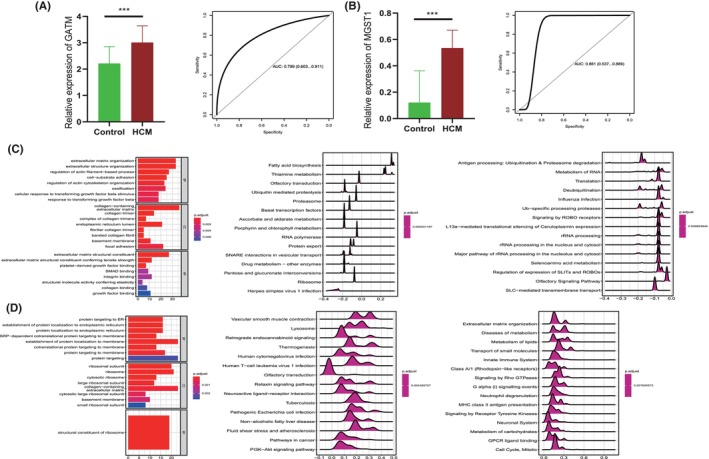
Validation of the different expression levels and diagnostic efficacies of GATM and MGST1 in our in‐house patients. (A) Barplot and ROC curve indicate different expression levels and diagnostic values of GATM and (B) MGST1. (C) Biological roles of GATM and MGST1 (D) based on association analysis, and the left to right shows GO, KEGG and GSEA results. ***p <0.001.

The different expression levels of GATM and MGST1 remain untested in tissues from our in‐house cohort. In addition, the underlying biological role of each gene remains unclear. Thus, we collected tissues of HCM and normal heart tissues after surgery and found that both GATM and MGST1 were more highly expressed in HCM (Figure 5A,B). Consistently, the diagnostic value of those two biomarkers exhibited high sensitivity and accuracy (AUC of GATM =0.79 and MGST1 = 0.88), which are shown in Figure 5A,B. The biological role of GATM might mainly involve extracellular matrix organization, collagen‐containing extracellular matrix and extracellular matrix structural constituent in GO terms; fatty acid biosynthesis, thiamine metabolism and ubiquitin‐mediated proteolysis in KEGG; and antigen processing and metabolism of RNA after GSEA (Figure 5C). Likewise, we found that MGST1 was mainly involved in protein targeting to ER, ribosomal subunit and structural constituent of ribosome in GO terms; vascular smooth muscle contraction, lysosome, retrograde endocannabinoid signalling in KEGG; and extracellular matrix organization, metabolism of lipids and transport of small molecules in GSEA (Figure 5D).

### 3.5 Immune infiltration difference between HCM and normal heart tissues

**FIGURE 6:**
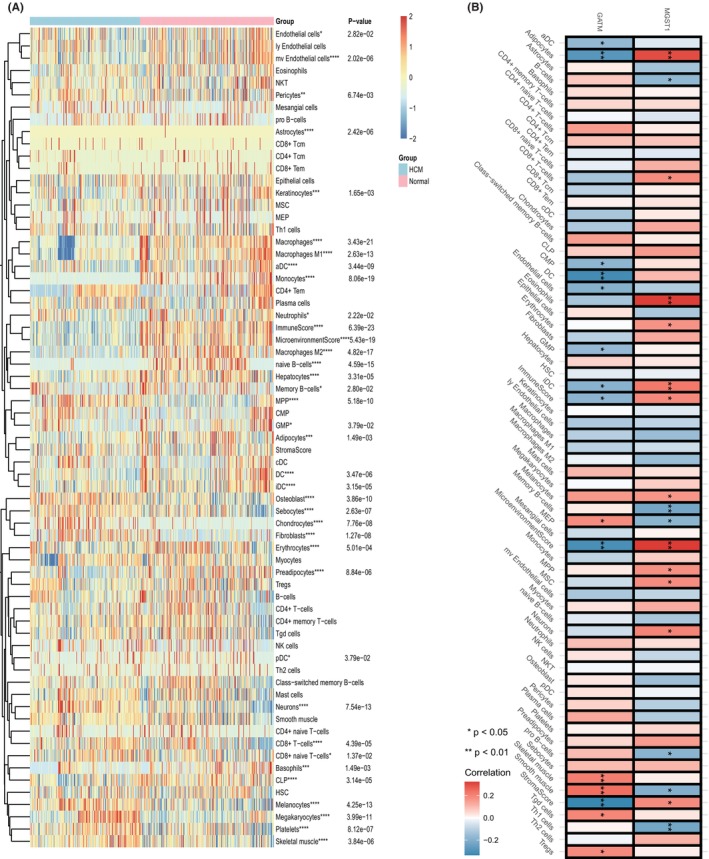
Immune cell infiltration landscape and correlation of biomarkers with immune signals based on the Xcell algorithm. (A) Heatmap showing different degrees of immune cell infiltration between HCM and normal heart tissues. (B) Correlation of GATM and MGST1 with immune cells and signals under the Xcell algorithm. *p <0.05, **p <0.01, ***p <0.001, ****p value <0.0001.

The results of DEG annotation reminded us that immune processes could alter the normal structure of the heart. We thus first applied the Xcell algorithm to compare different immune infiltration degrees between HCM and normal tissues and found a different immune landscape. In general, endothelial cells, pericytes, keratinocytes, macrophages (both M1 and M2), DCs, monocytes, neutrophils and naive B cells were more highly infiltrated in normal heart tissues, while MPPs, osteoblasts, sebocytes, chondrocytes, fibroblasts, CD8^+^ T cells, melanocytes, megakaryocytes and platelets were enriched in HCM (Figure 6A). Interestingly, we observed that GATM was negatively related to infiltration of several immune cell types (including DCs, adipocytes, CMPs, endothelial cells and GMPs) and immune‐related scores, while MGST1 displayed the reverse trend since it was positively related to adipocytes, CD8^+^ T cells, eosinophils, DCs and immune scores (Figure 6B). To further reveal which immune cell plays the leading role during HCM, we also applied the five machine learning algorithms mentioned previously and verified that monocytes and MPPs displayed pivotal roles in HCM (Figure S1A,B).

**FIGURE 7:**
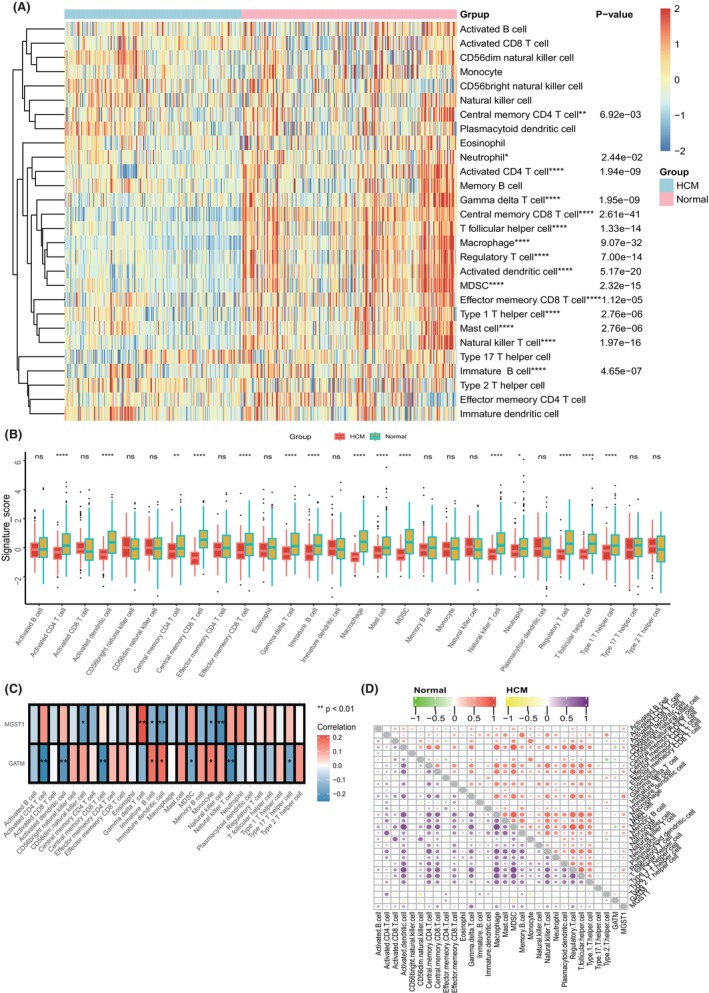
Immune cell infiltration landscape and correlation of biomarkers with immune cells based on the ssGSEA. (A) Heatmap and (B) boxplot indicate different immune cell infiltration degrees between HCM and normal heart tissues. (C) Correlation of GATM and MGST1 with immune cells and signals under the ssGSEA algorithm. (D) Correlation of immune cell infiltration suppression in normal and HCM tissues. ns, not significant, *p <0.05, **p <0.01, ***p <0.001, ****p <0.0001.

Consistently, we found that such immune differences were more obvious with the ssGSEA algorithm. As Figure 7A,B show, nearly all immune cells were highly infiltrated in normal heart tissues compared with HCM. GATM and MGST1 also led to different immune cell correlations under such an algorithm, since Figure 7C indicates that GATM was negatively correlated with activated CD4 T cells, activated dendritic cells, central memory CD8 T cells, mast cells and natural killer T cells but positively correlated with immature B cells, immature dendritic cells and monocytes. MGST1 was negatively correlated with CD56dim natural killer cells, immature B cells, immature dendritic cells, monocytes and natural killer cells but positively correlated with gamma delta T cells. However, the inner correlation of immune cells was consistent in both normal and HCM tissues (Figure 7D).

### 3.6 Overview of GATM and MGST1 in pancancer

**FIGURE 8:**
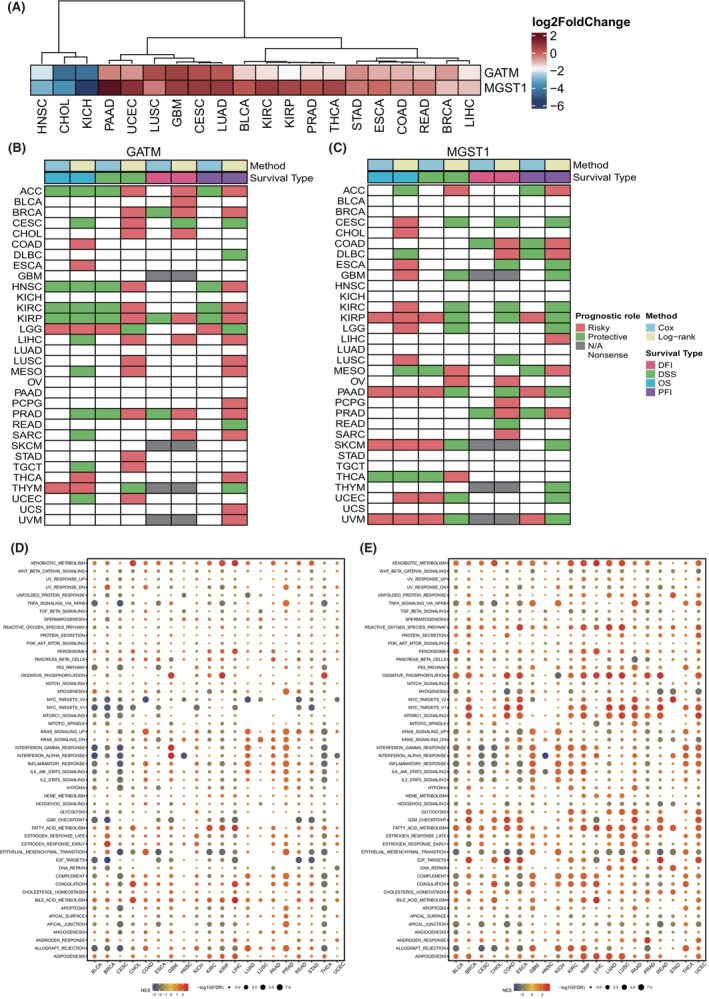
Comprehensive analysis of GATM and MGST1 across cancers. (A) Heatmap shows differential expression of GATM and MGST1 among cancers from the TCGA‐pancancer project. (B) Cox and log‐rank analyses of the prognostic value of GATM and MGST1 (C) from the perspectives of OS, DSS, DFI and PFI. (D) GSEA based on the top 25% of DEGs and the lowest 25% of GATM and MGST1 (E) in the HCM expression matrix.

Recently, several studies have suggested that pathogenesis of the heart might also affect cancer progression. Thus, we also investigated the role of GATM and MGST1 among cancers. Figure 8A shows that GATM and MGST1 were consistently upregulated in most cancer types except HNSC, CHOL and KICH. Interestingly, we noticed that GATM could influence the prognosis of ACC, HNSC, KIRC, KIRP, LGG and PRAD, while MGST1 could affect the prognosis of KIRP, PAAD, SKCM, THCA and UVM (Figure 8B,C). Furthermore, we found that GATM might mainly contribute to xenobiotic metabolism, MYC targeting, and epithelial mesenchymal transition among cancers, while MGST1 participated in xenobiotic metabolism, TNFa signalling, the reactive oxygen species pathway, oxidative phosphorylation, interferon, the G2/M checkpoint, and epithelial mesenchymal transition in cancers (Figure 8D,E). Interestingly, we noticed that both MGST1 and GATM were positively correlated with classic oncogenic signals including apoptosis, hypoxia, inflammation, metastasis and stemness (Figure S2A,B). GATM was significantly correlated with most immune checkpoint genes among cancers, which was extraordinarily obvious in PRAD, THCA and UVM. Similarly, we found that MGST1 played a pivotal immune role in LAML, LGG and TGCT (Figure S3A). In addition, the expression levels of GATM and MGST1 could be significantly influenced by DNA MMR deficiency, since their expression levels were correlated with five MMR genes, including MLH1, MSH2, MSH6, PMS2 and EPCAM (Figure S3B). DNA methylation might also impact the expression levels of GATM and MGST1 because four methyltransferases were significantly correlated with their expression among cancers (Figure S3C).

## 4 DISCUSSION

Cardiac ultrasound is the mainstay of clinical diagnosis of HCM, but it does not easily visualize the apex of the heart, which can lead to missed diagnoses in apical hypertrophic cardiomyopathies. With the development of modern cardiac imaging techniques such as cardiac magnetic resonance (CMR), which helps to identify more HCM phenotypes that were previously undetectable or easily missed by echocardiography, the global incidence of HCM is increasing at an alarming rate, estimated to be 1 in 200 (0.5%), and poses a challenge to the accurate management of HCM in clinical practice. The global incidence of HCM is increasing at an alarming rate, estimated to reach 1 in 200 (0.5%), posing a challenge to accurate management in clinical practice. Unfortunately, HCM frequently results in target organ damage that deteriorates the patient's prognosis, which is highly correlated with heart failure onset and progression.^27^ Despite the discovery of myotubular genes, no other major causal genes have been identified for HCM as a monogenic disease.^28^ In contrast, HCM may potentially result from the synergistic effect of multiple genetic variants. Previous studies showed that certain genetic variants could contribute to HCM development and alter disease phenotype and prognosis.^29^, ^30^ However, these findings remain limited to small populations and family lines, and large cohort results are lacking. Thus, the pathogenesis of HCM is still unknown, and few effective treatments are available. As a result, it is crucial to improve our comprehension of the underlying mechanisms of HCM and identify potential therapeutic targets.

In this work, we performed a systematic analysis to identify novel biomarkers for HCM at the expression level. Four HCM datasets were integrated as the training cohort, and one independent dataset was treated as the testing cohort. We used five machine learning algorithms with good performance to extract signatures relevant to HCM. Then, two robust biomarkers (GATM and MGST1) were identified, and the combination of the two biomarkers was applied to construct a novel risk system for HCM. We also assessed the diagnostic role of immune components via the Xcell algorithm and found that MPP and monocytes played important roles in HCM. In addition, HCM contained a relevant exclusion immune state compared with normal tissues. Finally, we used a multiomics dataset of cancer from the TCGA portal and found a potential correlation between HCM and cancer progression. Our study is the first to investigate biomarkers with the application of multiple cohorts.

It should be mentioned that all those biomarkers of HCM found in previous works were only identified by a single machine learning algorithm or only based on different expression or WGCNA, which might attenuate the robustness and sensitivity. In contrast, biomarkers filtered through a crossoverlap analysis have the potential to enhance our understanding of the pathophysiology of HCM and assist in the diagnosis and risk stratification of this disease.^4^ Several biomarkers have been studied in relation to HCM, including natriuretic peptides, troponins, cytokines and microRNAs. Zhao and colleagues found four biomarkers of HCM, including S100A8, S100A8, TYROBP and FCER1G, which are related to M1 proinflammatory cells.^31^ Li et al. found that two m6A readers, YTHDC1 and IGFBP3, could distinguish HCM and affect the immune landscape and energy metabolism‐related pathways in HCM tissues.^32^ Yu and colleagues applied different expression and WGCNA analyses and found that MAFB, MT1M and LYVE1 might be novel key genes affecting the inflammatory response in HCM.^33^ However, the datasets enrolled in this work only contained limited datasets, and the number of this work was not suitable for machine learning, which might cause false positive events to some extent. In contrast, biomarkers (GATM and MGST1) and the corresponding risk score system were not reported by previous works, which might be accounted for by the study design and application of multimachine learning algorithms. While these biomarkers hold promise for improving the care of patients with HCM, further research is needed to establish their clinical utility and validate their use in routine clinical practice.

In addition, several novel biological processes and pathways that might also be involved in HCM were also found in our work. In addition to classic signals, including cellular divalent inorganic cation homeostasis and channel‐related complexes, we confirmed that some immune and metabolism pathways, such as cytokine interactions and tyrosine metabolism, might also function in HCM. Previous studies have suggested that alterations in threonine and serine phosphorylation levels are hallmarks of heart failure. Xu and colleagues applied two models of cardiac hypertrophy and proved that tyrosine phosphorylation plays a pivotal regulatory role in HCM.^9^ A study from Schuldt et al. also found that the detyrosination effect of microtubules could trigger the pathogenesis of HCM with sarcomere mutation‐positive characteristics.^34^ All these studies proved that specific amino acid metabolism could contribute to the occurrence and progression of HCM. In addition, glycine, serine, thionine, taurine and homotaurine might be treated as therapeutic approaches for HCM patients. For immune landscape differences between HCM and normal tissues, we introduced five machine learning algorithms and found the two most important immune components involved in HCM. Previous works have revealed that monocytes in myocardial infarction could recruit monocytes into heart tissues via a myeloid differentiation primary response 88‐dependent approach, which facilitated myocardial remodelling.^35^, ^36^, ^37^ In addition to macrophages, MPP might function as another important immune component involved in HCM, and the detailed roles of MPP need to be investigated in the future.

Cardiovascular disease (CVD) and cancer are significant public health concerns that remain the top two leading causes of mortality worldwide. Conventionally viewed as distinct entities, there is mounting evidence illustrating common risk factors and similar pathogenesis between CVD, particularly heart failure, and cancer, which mutually influence each other.^38^ Clinical epidemiological data reveal a higher likelihood of cancer incidence among individuals with CVD than among those without CVD, with risk proportionate to greater CVD risk factor exposure.^39^ Myocardial remodelling or heart failure has been shown in several preclinical studies to significantly stimulate tumour growth acceleration, compellingly demonstrating the causal relationship between these conditions.^40^ Work from Yang et al. reminded us that myocardial exosomes from ischemic cardiomyocytes could transport mature miR‐22‐3P into tumour cells, thus repressing the normal expression level of the ferroptosis sensitivity‐related regulator ACSL4.^41^

In the complex landscape of disease interplay, the association between CVD and cancer emerges as both pivotal and profound. Recent insights suggest a bidirectional relationship where not only do share risk factors such as inflammation, obesity and smoking contribute to both ailments, but the pathophysiological processes underlying CVD may directly influence cancer progression.^42^ The development and progression of atherosclerosis, for instance, involve inflammatory processes that can potentially support the oncogenic pathways. Atherosclerotic changes in the vascular environment may alter the local and systemic milieu in ways that could foster cancer cell survival, proliferation and metastasis. Conversely, the systemic effects of cancer, including the release of pro‐inflammatory cytokines and growth factors, could exacerbate cardiovascular complications. This interaction is evidenced by studies showing that cardiovascular impairments like heart failure and myocardial infarction can accelerate tumour growth and metastatic spread, likely through mechanisms involving immune system modulation and inflammatory response amplification.^43^ Furthermore, treatments for each condition often intersect, with cardiotoxic effects being a notable concern in cancer therapy, such as the use of anthracyclines and HER2 inhibitors in oncology that necessitate careful cardiovascular monitoring. Conversely, cardiovascular drugs like statins and beta‐blockers show potential in reducing cancer risk or mitigating its progression, hinting at shared molecular pathways that could be therapeutically exploitable.^41^ These observations underscore the necessity for a holistic view in patient care, emphasizing that the management of patients with either CVD or cancer should always consider the potential for comorbid conditions. The integration of cardio‐oncology as a subspecialty is a testament to the evolving understanding of the interdependence between these two fields, aiming to optimize treatment outcomes by addressing the cardiovascular risks in cancer patients and vice versa. In this work, when we deciphered the role of GATM and MGST1 at the pancancer level, we found that the two signatures displayed similar expression, prognostic, and biological influence patterns. A previous study revealed that GATM functions as a downstream factor in promoting the progression of FLT3‐ITD‐mutant acute myeloid leukaemia by regulating de novo creatine biosynthesis.^44^ In addition, as a glycine amidinotransferase, Gatm in tumour‐adjacent adipose tissue could accelerate breast cancer metastasis by interacting with Acsbg1.^45^ Jee et al. revealed that GATM could be treated as a tumour suppressor by enhancing the role of PBRM1 in ccRCC followed by immune checkpoint therapy.^46^ In a pivotal study, Yang et al. employed comprehensive multi‐omics techniques, including ChIP‐qPCR, 3C‐qPCR and CRISPRi/dCas9, to explore the role of GATM in regulating guanidinoacetic acid, which in turn enhances cell migration and epithelial‐mesenchymal transition (EMT).^47^ This process is mediated through the modulation of c‐Myc‐driven expression of high mobility group AT‐hook proteins. Importantly, they demonstrated that guanidinoacetic acid augments the H3K4me3 modification level via upregulation of specific histone methyltransferases, thereby promoting the transcription of genes linked to metastasis, including Myc. Further extending these findings, Zhang et al. uncovered that GATM‐mediated creatine synthesis can potentiate cancer metastasis and diminish survival in mice.^48^ This effect is driven by the upregulation of Snail and Slug expression, facilitated by monopolar spindle 1 (MPS1)‐activated phosphorylation of Smad2 and Smad3. Consequently, targeting GATM or MPS1 presents a promising therapeutic strategy to inhibit cancer metastasis, particularly in colorectal cancers harbouring mutations in the transforming growth factor beta receptor. These studies collectively underscore the multifaceted role of GATM in the cancer ecosystem, corroborating the expression and prognostic analyses presented in our study. Similarly, existing research highlights MGST1 as a critical player in various cancers, reinforcing its potential as a therapeutic target. Kuang found that high expression levels of MGST1 in pancreatic cancer cells might bind to ALOX5, further reducing lipid peroxidation.^49^ Another study also investigated the progression of lung adenocarcinoma by activating AKT/GSK‐3β signalling and regulating apoptosis via mitochondrial pressure. According to previous works and the novel findings in our work, we hypothesized that high GATM and MGST1 expression in HCM might promote cancer progression through xenobiotic metabolism and immune‐related signatures, and the detailed cause‐result relation needs to be further investigated.

In our work, we have employed a multimachine learning algorithm‐based pipeline to identify novel diagnostic and therapeutic targets for HCM, presenting potentially groundbreaking avenues for future research. However, several limitations are inherent to our approach that warrant further exploration to enhance the robustness and clinical applicability of our findings. Firstly, our study's retrospective design necessitates validation through prospective cohorts to test the accuracy and sensitivity of the identified biomarkers, GATM and MGST1, and to evaluate the performance of the proposed nomogram. Prospective validation would not only confirm these biomarkers' utility but also refine their diagnostic thresholds which are crucial for clinical applications. It is important to note that the cutoff values for GATM and MGST1 may vary based on ethnic backgrounds and the type of diagnostic equipment used, which could introduce variability in their diagnostic accuracy. Therefore, substantial preclinical trials are required to quantify these biomarkers' diagnostic values accurately under diverse clinical settings. Secondly, while our study has utilized arrays predominantly, future studies should consider incorporating analyses of noncoding RNAs and methylation patterns, which could provide deeper insights into the genomic underpinnings of HCM. Such inclusion could potentially unveil additional layers of regulatory mechanisms influencing HCM pathophysiology. Thirdly, the adoption of four machine learning algorithms in our study has set a foundational framework for biomarker identification. Nevertheless, incorporating more advanced and possibly hybrid machine learning algorithms in future research could further enhance the identification process, offering more sophisticated tools to dissect the complex biological datasets. Lastly, the biological roles of GATM and MGST1 need extensive molecular biology validation to elucidate their functional mechanisms comprehensively. Follow‐up studies involving knockdown experiments and exploration of downstream effects are critical to understand how these biomarkers influence HCM at a molecular level. Such studies will not only validate the roles of these biomarkers but also could lead to the discovery of novel therapeutic targets, thereby opening new avenues for treating HCM.

In summary, we identified and verified two promising biomarkers involved in HCM that might be treated as novel targets. We also found that HCM led to an obvious immune dysregulation state compared with normal heart tissues, and two important immune components, MPP and monocytes, might aid HCM progression. A brief and robust risk system was also constructed and tested in our work and could serve as a novel risk stratification for HCM. In addition, we revealed that GATM and MGST1 played significant roles in cancer by affecting several classic cancer hallmarks.

## AUTHOR CONTRIBUTIONS

**Hualei Dai:** Data curation (equal); resources (equal). **Ying Liu:** Data curation (equal); formal analysis (equal). **Meng Zhu:** Writing – original draft (equal); writing – review and editing (equal). **Siming Tao:** Data curation (equal); formal analysis (equal). **Chengcheng Hu:** Data curation (equal); software (equal). **Peng Luo:** Conceptualization (equal); methodology (equal). **Aimin Jiang:** Conceptualization (equal); investigation (equal). **Guimin Zhang:** Conceptualization (equal); project administration (equal).

## FUNDING INFORMATION

This work is supported by the Association Foundation Program of Yunnan Provincial Science and Technology Department and Kunming Medical University (nos. 202001AY070001‐087, 202001AY070001‐243), the Cardiovascular Ultrasound Innovation Team of Yunnan Province (no. 202305AS350021) and the High‐Level Talent Training Support Plan of Yunnan Province(nos. YNWR‐MY‐2018‐001, YNWR‐MY‐2020‐024).

## CONFLICT OF INTEREST STATEMENT

The authors declare no conflict of interest.

## CONSENT

Not applicable.

## Supporting information


**Figure S1.** Most important immune cells involved in HCM based on machine learning. (A) The interaction of five machine learning methods to identify the most important immune cell signals. (B) ROC curve indicating the diagnostic value of monocytes and multipotent progenitors (MPPs) in the training and testing (C) cohorts.

**Figure S2.** Correlation between expression level of GATM (A) and MGST1 (B) and enrichment score of classic oncogenic signals.

**Figure S3.** Correlation of GATM and MGST1 expression and immune signatures, DNA mismatch repair genes and methyltransferases in pancancer. (A) Heatmaps show the expression correlation of GATM and MGST1 expression levels with immune signatures and MMR (B) among cancers. (C) Circle heatmap reveals the correlation of GATM and MGST1 expression and four methyltransferases.

## Data Availability

All datasets utilized in this study can be found in the Section 2.
