# Supplementary figures and images for: Machine learning and experimental validation of novel biomarkers for hypertrophic cardiomyopathy and cancers
Source: J Cell Mol Med. 2024 Aug 19;28(16):e70034. doi: 10.1111/jcmm.70034 (PMC11333198; doi:10.1111/jcmm.70034)

A

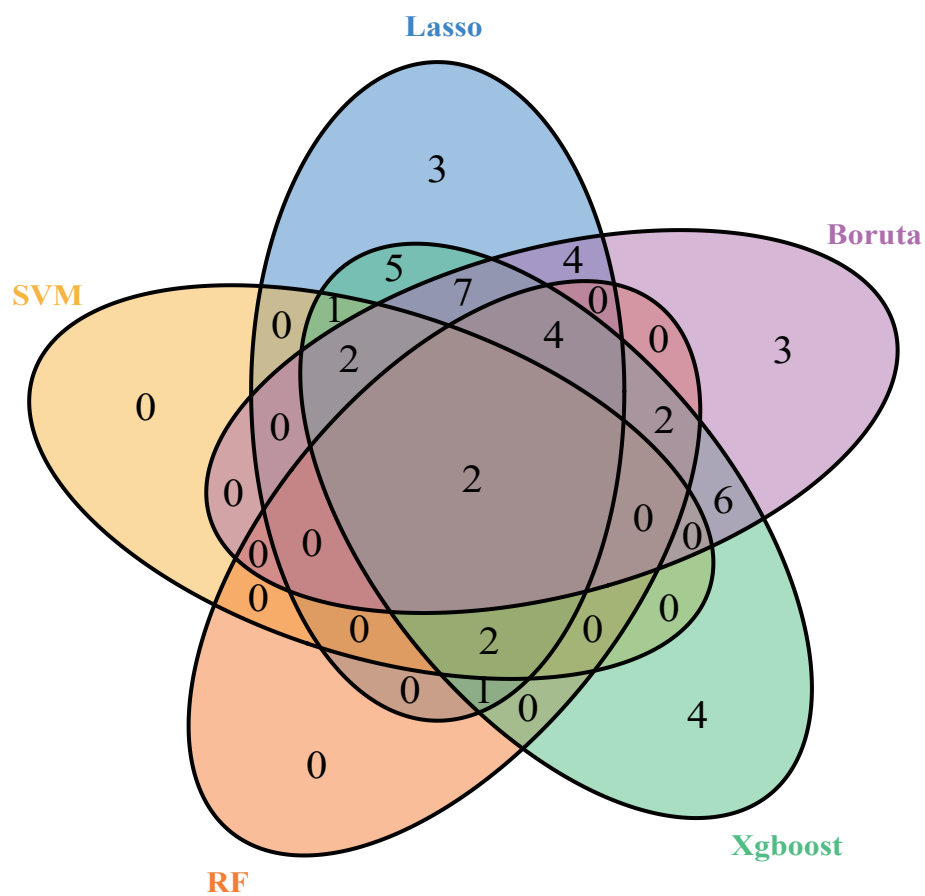

B

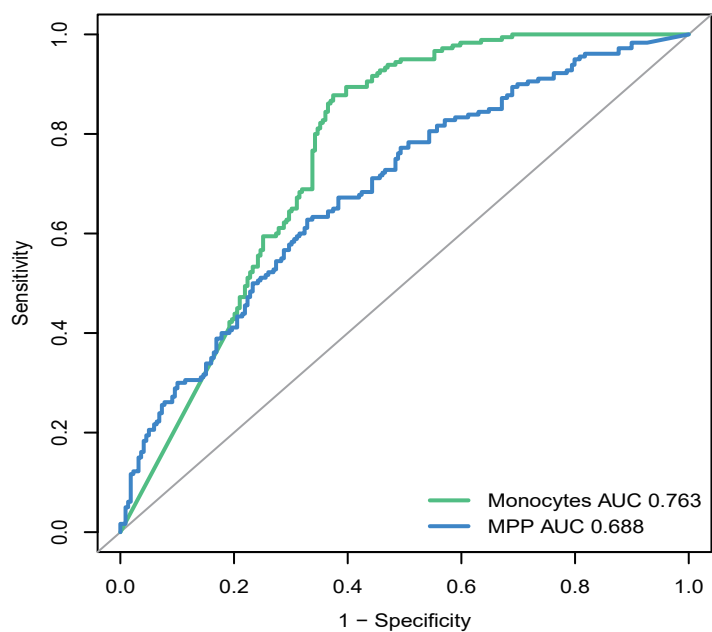

C

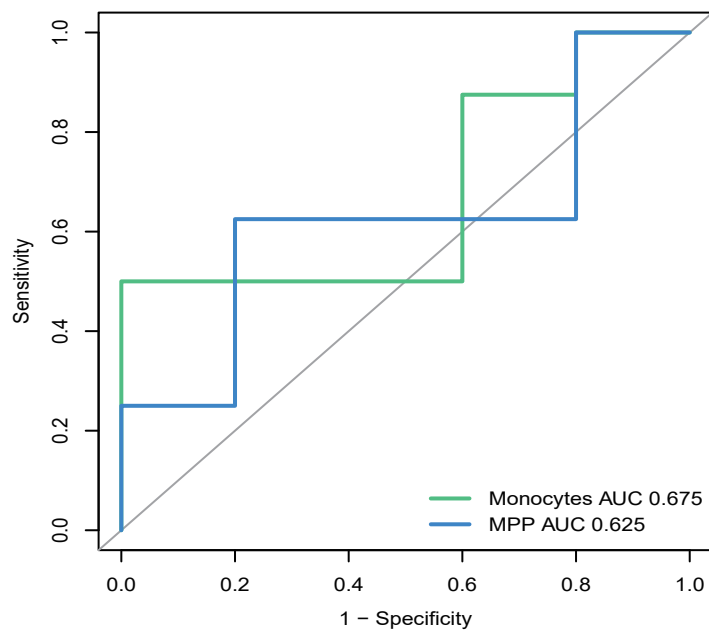

Supplement: Supplementary file 1 — Figure S1. Most important immune cells involved in HCM based on machine learning. (A) The interaction of five machine learning methods to identify the most important immune cell signals. (B) ROC curve indicating the diagnostic value of monocytes and multipotent progenitors (MPPs) in the training and testing (C) cohorts. [file JCMM-28-e70034-s002.pdf]

A

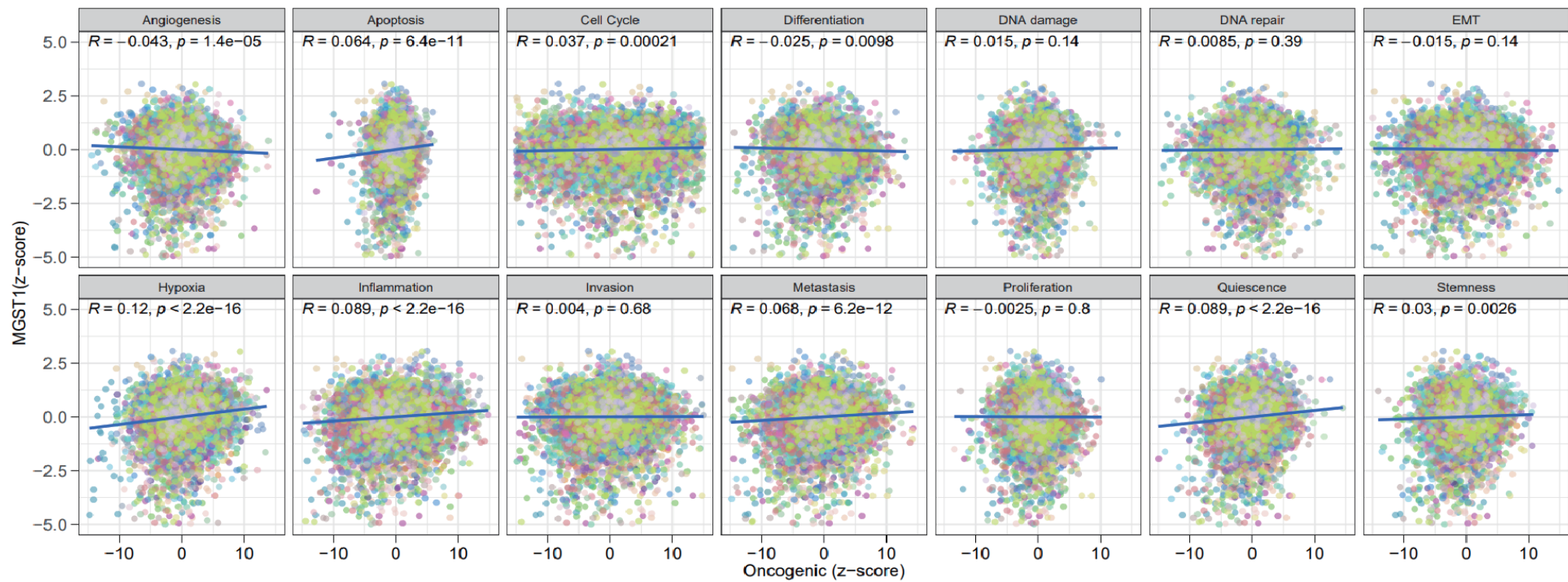

B

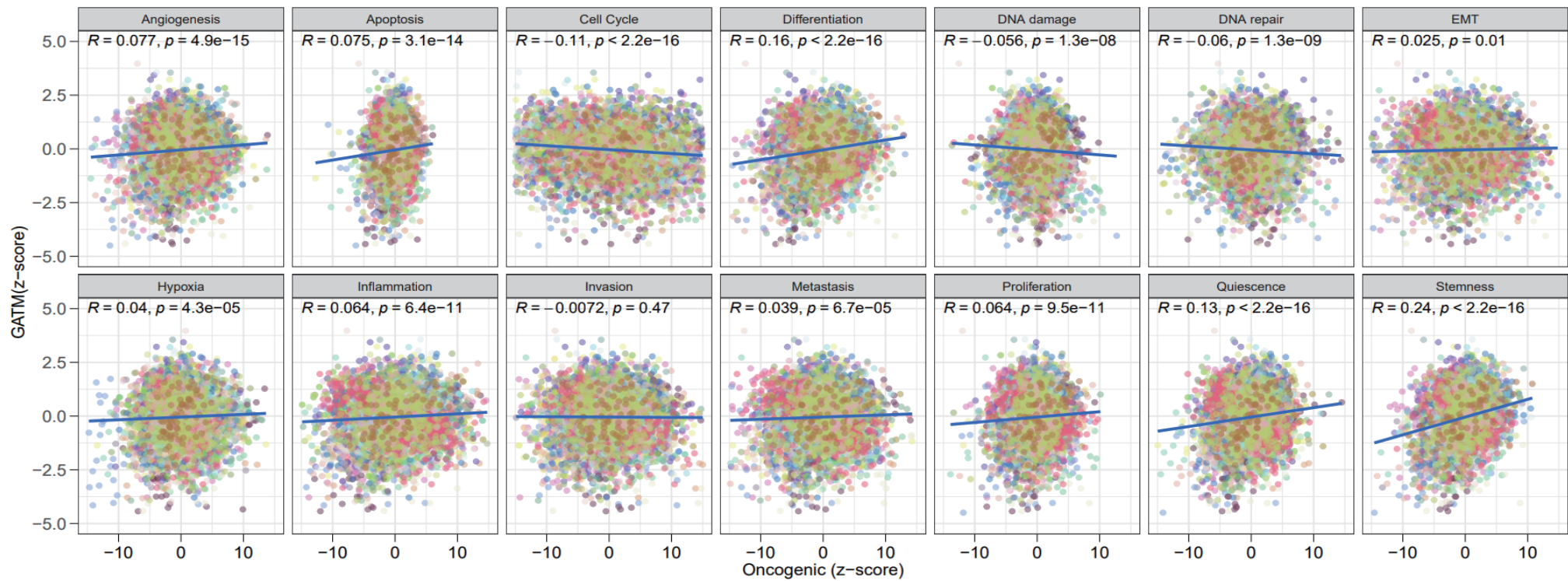

Supplement: Supplementary file 2 — Figure S2. Correlation between expression level of GATM (A) and MGST1 (B) and enrichment score of classic oncogenic signals. [file JCMM-28-e70034-s001.pdf]

A

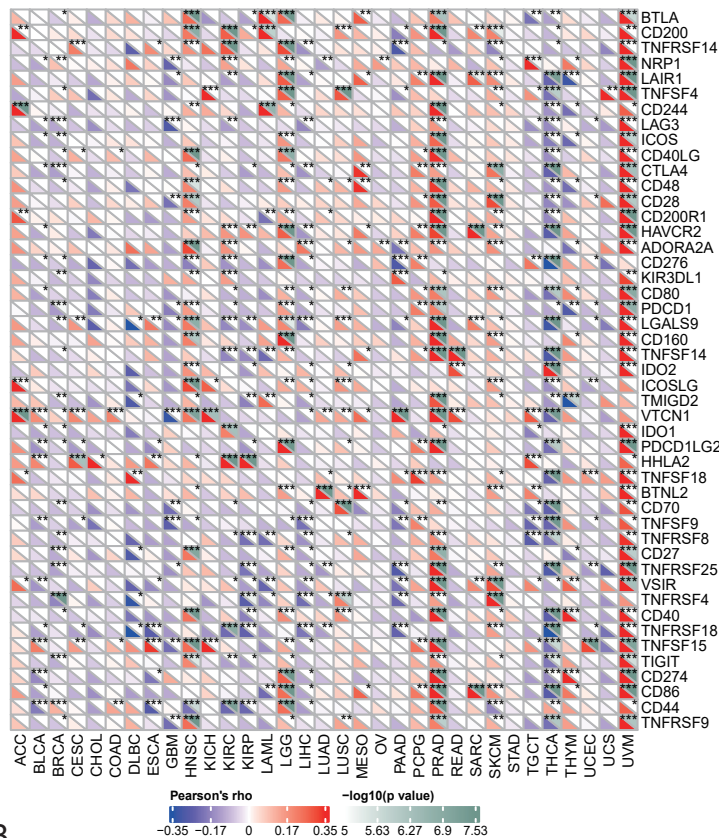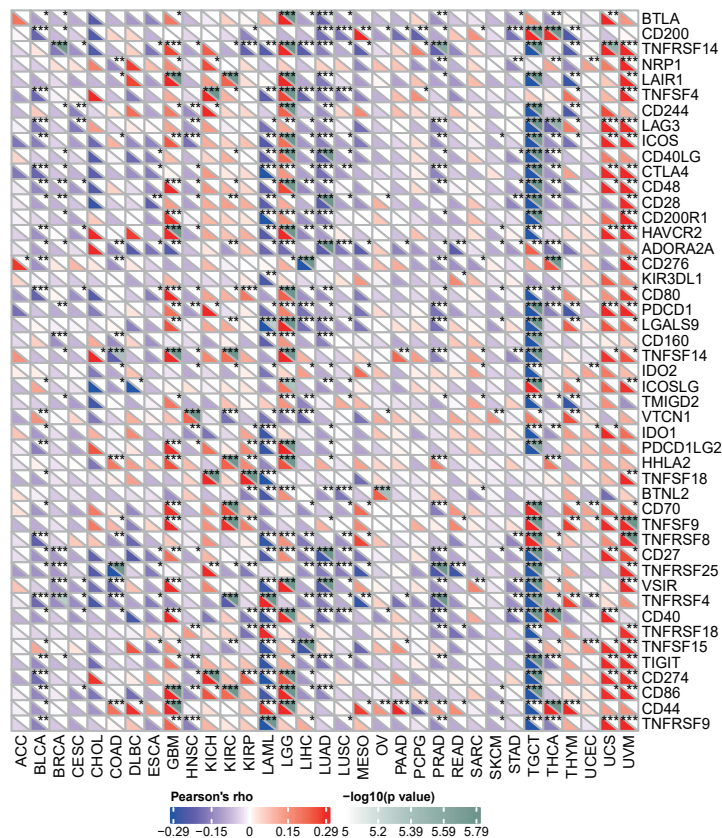

B

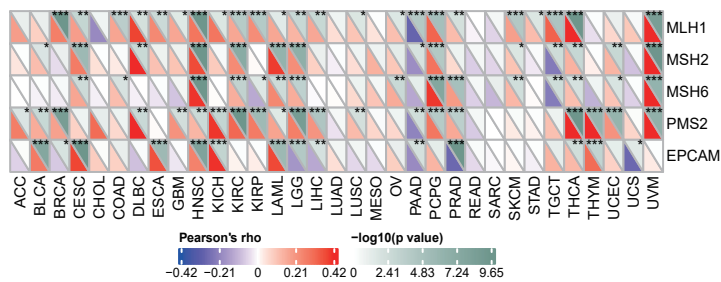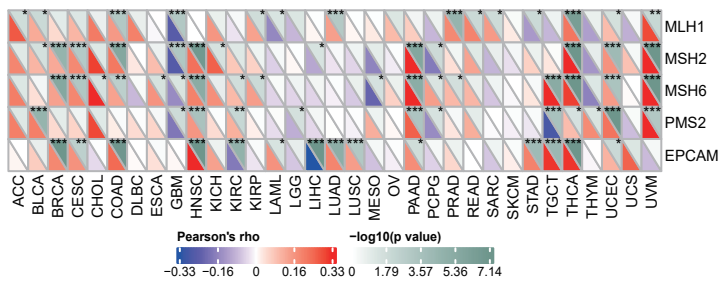

C

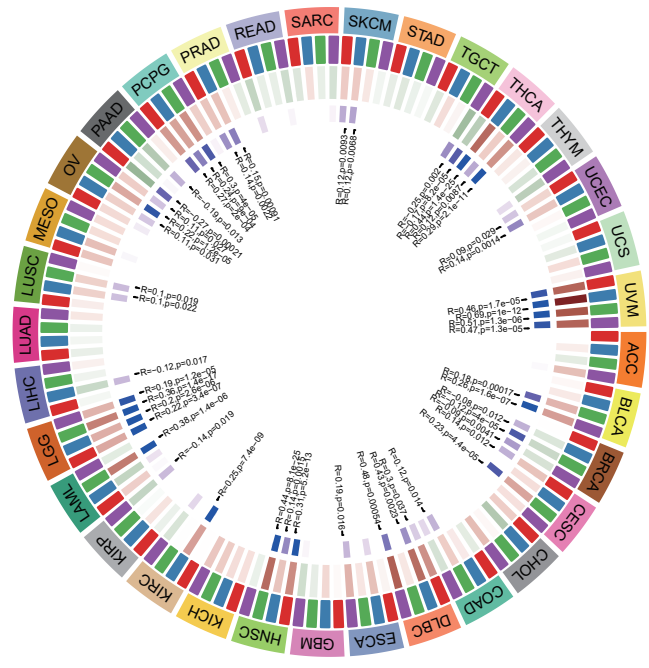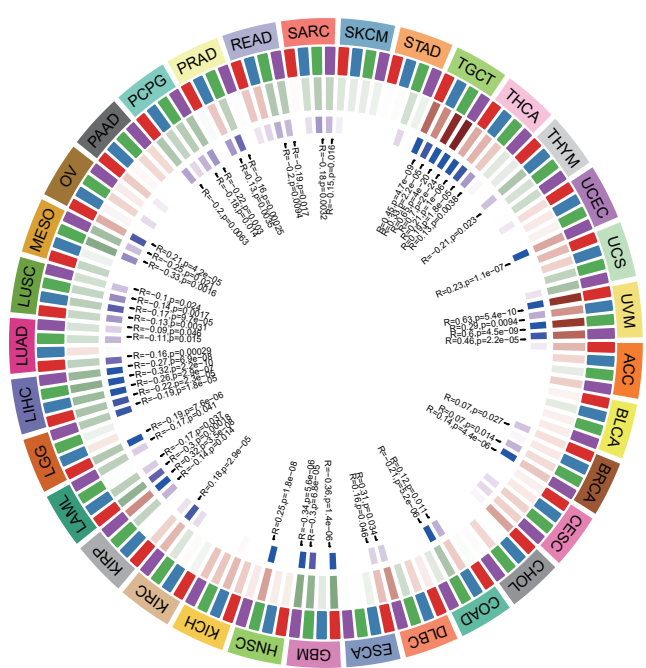

Supplement: Supplementary file 3 — Figure S3. Correlation of GATM and MGST1 expression and immune signatures, DNA mismatch repair genes and methyltransferases in pancancer. (A) Heatmaps show the expression correlation of GATM and MGST1 expression levels with immune signatures and MMR (B) among cancers. (C) Circle heatmap reveals the correlation of GATM and MGST1 expression and four methyltransferases. [file JCMM-28-e70034-s003.pdf]
